# Accuracy of Body Mass Index Cutoffs for Classifying Obesity in Chilean Children and Adolescents

**DOI:** 10.3390/ijerph13050472

**Published:** 2016-05-05

**Authors:** Rossana Gómez-Campos, Raquel David Langer, Roseane de Fátima Guimarães, Mariana Contiero San Martini, Marco Cossio-Bolaños, Miguel de Arruda, Gil Guerra-Júnior, Ezequiel Moreira Gonçalves

**Affiliations:** 1 Instituto de Actividad Física y Salud, Universidad Autonoma de Chile, 5 Poniente 1670, Talca, Chile; 2 Faculty of Physical Education, State University of Campinas, Avenida Érico Veríssimo, 701, Cidade Universitária Zeferino Vaz, Barão Geraldo, CEP 13083-851, Campinas, Brazil; mcossio1972@hotmail.com (M.C.-B.); miguelfef@hotmail.com (M.d.A.); 3 Department of Research, Universidad Científica del Sur, Panamericana Sur Km 19 Villa, Lima, Perú; 4 Growth and Development Lab, Center for Investigation in Pediatrics (CIPED), School of Medical Sciences, University of Campinas (UNICAMP), CEP 13083-887, Campinas-SP, Brazil; raqueldlanger@gmail.com (R.D.L.); roseanefguimaraes@gmail.com (R.d.F.G.); mari_martini08@yahoo.com.br (M.C.S.M.); gilguer@fcm.unicamp.br (G.G.-J.); emaildozeique@gmail.com (E.M.G.); 5 Department of Physical Activity Sciences, Catholic University of Maule, Av. San Miguel 3605, Talca, Chile; 6 Research Network on Human Biological Development, Urb. Amauta C-6, Jose Luis Bustamante y Rivero, Arequipa, Peru

**Keywords:** Body Mass Index, obesity, DXA, cut-off points, Chile

## Abstract

*Objective*: To determine the accuracy of two international Body Mass Index (BMI) cut-offs for classifying obesity compared to the percentage of fat mass (%FM) assessed by Dual-Energy X-ray Absorptiometry (DXA) in a Chilean sample of children and adolescents; *Material and Methods*: The subjects studied included 280 children and adolescents (125 girls and 155 boys) aged 8 to 17 years. Weight and height were measured. The BMI was calculated. Two international references (IOFT and WHO) were used as cut-off points. The %FM was assessed by DXA. The receiver operating characteristic (ROC) curve was used to assess the performance of BMI in detecting obesity on the basis of %FM; *Results*: A high correlation was observed between the %FM measured by the DXA and the Z-scores of IOTF and WHO scores in the Chilean adolescents separated by sex (*r* = 0.78–0.80). Differences occurred in both references (IOFT and WHO) in relation to the criteria (*p <* 0.001). Both references demonstrated a good ability to predict sensitivity (between 84% and 93%) and specificity (between 83% and 88%) in both sexes of children and adolescents; *Conclusions*: A high correlation was observed between the Z-score of the BMI with the percentage of fat determined by the DXA. Despite this, the classifications using the different BMI cut-off points showed discrepancies. This suggests that the cut-off points selected to predict obesity in this sample should be viewed with caution.

## 1. Introduction

The prevalence of obesity is increasing at an alarming rate in many countries around the world [1]. Moreover, a dramatic rise in obesity has also occurred in all ages including adolescents in Latin America [2]. Obesity is responsible for the increase in risks associated with morbidity such as those linked to cardiovascular disease, diabetes, arterial hypertension, dyslipidemia, and accidents caused by ischemic strokes [3]. Moreover, obesity has been linked to the increased risk of various types of cancer resulting in the death of approximately 28 million persons annually worldwide [4]. In Chile, childhood obesity has increased substantially almost doubling in 22 years rising from 8.7% in 1989 to 23.1% in 2010 [5].

In children and adolescents, obesity leads to innumerable emotional, social, and health problems causing concern for the implications for these future generations [6]. In addition, obesity contributes to diverse metabolic risk factors that may worsen in adulthood [7,8]. Therefore, infancy and adolescence are essential stages in the search for the prevention of obesity since these are the ages characterized by biological, psychological, and cognitive changes [9] that occur during these phases of life.

Due to the simplicity of the measurement, the Body Mass Index (BMI) is a widely acceptable method for the determination of obesity in epidemiological studies [10,11]. Among children and adolescents, two worldwide BMI criteria are used to access obesity: the cutoffs proposed by the International Obesity Task Force (IOTF) and the World Health Organization (WHO). These references highlight the use of representative samples of different ethnicities. The IOTF [12] is based on six large international representative data sets (Brazil, China, the United States, Great Britain, Holland, and Singapore). The WHO [13] data set was created using information from the 1997 National Center for Health Statistics–NCHS (from 1 to 24 years) in conjunction with information from preschoolers less than 5 years old from 2006 collected by the WHO [14]. The WHO [13] system defines obesity as a BMI > 2 SD from the mean of the WHO reference population. The IOTF cutoff is an extrapolation of the adult BMI cutoff points for obesity (30 kg/m^2^) [12].

Different cutoff points are suggested for obesity diagnosis. However, several aspects should be considered before selecting the criteria. These should include cultural and ethnic characteristics. Additionally, BMI is not able to distinguish the body composition components for determining obesity. Differently Dual-Energy X-ray Absorptiometry (DXA) is considered to be a precise and accurate method to determine body composition [15]. Moreover, it is a fast, safe, and non-invasive method. It has the advantage of dividing the total mass into three categories: components (fat, mineral content, and mass free from fat and bone), total form (total body), and body segments (head, arms, legs, and trunk) [16]. Based on this perspective, the purpose of this study was to determine the ability of the IOTF and WHO BMI cutoffs to identify obesity in a sample of Chilean children and adolescents compared to the values of the percentage of fat mass (%FM) determined by the DXA.

## 2. Methods

### 2.1. Sample

This was a cross-sectional study. A total of 280 children and adolescents (125 girls and 155 boys) aged 8–17 years were include in this research. The subjects were selected from three public schools in the city of Talca (Chile). Talca is the capital of the Maule Region (Chile). It is located in the central valley of the Maule Region 110.5 m above sea level. It is 252 kilometers south of Santiago (the capital of Chile).The average age of boys was (*X* = 11.3 ± 2.02 years) and girls (*X* = 11.07 ± 2.01). Prior to the research, all parents and those responsible for the students met with the researchers and were provided with detailed information about the experiment. Then, they signed the informed consent forms granting permission for their children to participate. All students registered in their respective schools and those having parental consent were included in the study. Those students not showing up with the appropriate clothing the day the anthropometric measurements and DXA scanning were to be taken, or those with some type of metal implant and/or prosthetic were excluded from the study as were those not having informed parental consent. The study was approved by the ethics committee from the Universidad Autónoma de Chile (238/2013).

### 2.2. Data Collection

All data collection procedures were conducted in a standard laboratory with a temperature of 22–24 °C. The anthropometric measurements and the Dual-Energy X-ray Absorptiometry (DXA) were conducted from 8:30 to 14:00 during the months of October and November of 2014. Data collection was conducted by those professionals skilled and trained in taking anthropometric measurements and in the DXA scanning process. The first step of the process was to fill out forms with the students’ information (day, month, and year of birth). This information was provided by the school administrations. Next, the anthropometric measurements were taken, and, finally, the scan was performed.

All anthropometric measurements were performed by the same trained researcher in accordance with the International Society for the Advancement of Kinanthropometry [17]. Height was measured using a Standiometer (Seca: Hamburg, Germany) graduated in millimeters with a precision of 0.1 cm. Weight was evaluated with a weighing-scale (Tanita Ltd.: Tokyo, Japan) with children and adolescents wearing only underwear. BMI was calculated as weight (kg) divided by height (m^2^).

Whole-body DXA scans were performed to assess the %BF according to the procedures recommended by the manufacturer on a fan beam (Lunar Prodigy; General Electric: Fairfield, CT, USA). To verify the viability of the equipment, 10% of the sample (33 subjects) was scanned twice. These showed a Technician Measurement Error (TME) of less than 1.5%.

### 2.3. BMI Classification and Outcome Variables

BMI was calculated for all subjects classified as overweight, obese, or neither overweight nor obese according to the IOTF, and WHO cutoffs. IOTF cut-off are an extrapolation of the adult BMI cut-off points for overweight (25 kg/m^2^) and obese (30 kg/m^2^). The WHO [18] system defines overweight as a BMI > 1 SD and obesity as a BMI > 2 SD, corresponding to 97.7 percentile from the WHO reference population [13].

### 2.4. Classification of the Z-Score

The BMI values were transformed into the Z-score using the formula below:

$$Z=\frac{{\left(\frac{BMI}{M}\right)}^{L}-1}{L\times S}$$

The values of L, M, and S refer to age and sex of each child and adolescent in keeping with the references in the literature: IOTF [12], WHO [13], and NHANES [19]. The values of the %FM measured by DXA were classified using the NHANES [19] reference. This reference includes children beginning at 8 years of age.

### 2.5. Statistical Analysis

The Shapiro Wilk’s test was used to verify the normality of the data. The data did not show a normal distribution. As a result, non-parametric tests were used. These were represented in the minimum and maximum median. The Spearman test was used to determine the correlational coefficient (*r*). The figures depicted here were created using SigmaPlot for Windows version 11.0 software (Systat Software Inc.: Erkrath, Germany). The Chi^2^ test was calculated to determine the association between the classifications of the variables. Receiver operating characteristic (ROC) curve was used to assess the performance of BMI in detecting obesity on the basis of %FM. Cut off points for specificity and sensitivity percentages of BMI in detecting obesity were calculated on the basis of %FM by DXA. We define excess fatness (obesity) the values of %FM by DXA as above the 90th percentile according to sex and age [19]. The area under the ROC curve (AUC) was determined to provide a numerical summary of the indicator’s performance. The Receiver Operating Characteristic (ROC curve) were generated to analyze the best cut-off points for the BMI to detect obesity starting from the values obtained from the DXA. Data was significant when *p <* 0.05. SPSS version 18.0 software (IBM: Chicago, IL, USA) was used to analyze all data.

## 3. Results

The characteristics of the children and adolescents participating in the study are presented by sex in Table 1 below.

Table 2 shows the analysis for both sexes of Chi^2^ using the %FM measured by the DXA. Also, based on sex, the table depicts the Z-score of the BMI based on the references from the IOTF [12] and the WHO [13]. In the sample of boys (*n* = 155), 23 were classified as obese based on the NHANES [19], 14 on the IOTF [12], and 21 on the WHO [13] standards. Using the NHANES [19], 27 girls (*n* = 125) were classified as obese. Based on the IOTF [12] and the WHO [13] references, 18 and 22 respectively were classified as obese.

**Figure 1:**
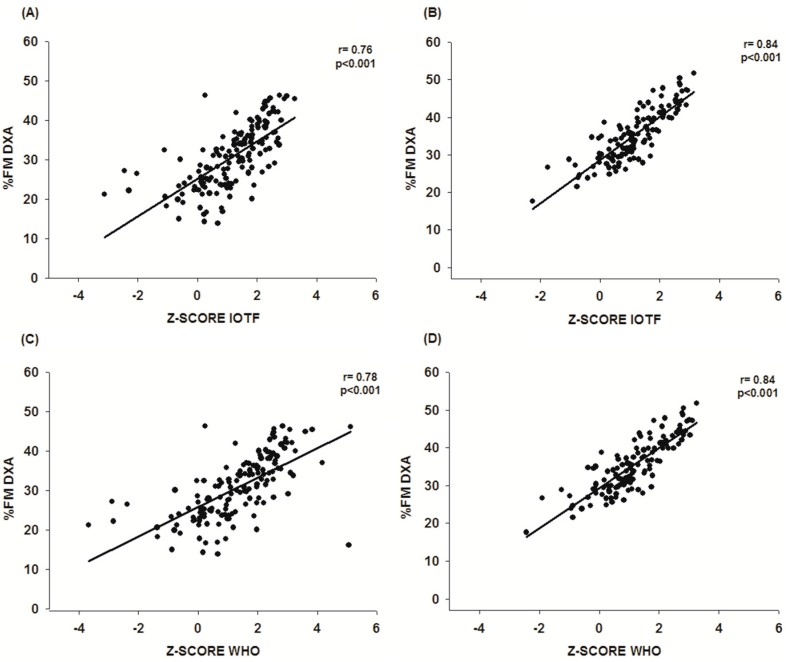
Correlation between the %FM measured by the DXA and the Z-score: boys (**A**) and girls (**B**) classified by the IOTF [12]: and boys (**C**) and girls (**D**) classified by the WHO [13] in the sample of Chilean children and adolescents.

Figure 1 illustrates the correlation between the %FM measured by DXA and the Z-scores IOTF [12] and the WHO [13] of Chilean children and adolescents based on sex. In the sample of boys and girls for this study, the correlation between the %FM measured by DXA and the Z-scores results are in keeping with the classification by Cole and Lobstein [12]. For boys, it was (*r* = 0.78). For the girls, the correlation was higher: (*r* = 0.80), *p <* 0.001. The correlation between %FM measured by DXA and the BMI Z-score based on the WHO [13] classification was strong for both sexes: (*r* = 0.80), *p <* 0.001.

**Figure 2:**
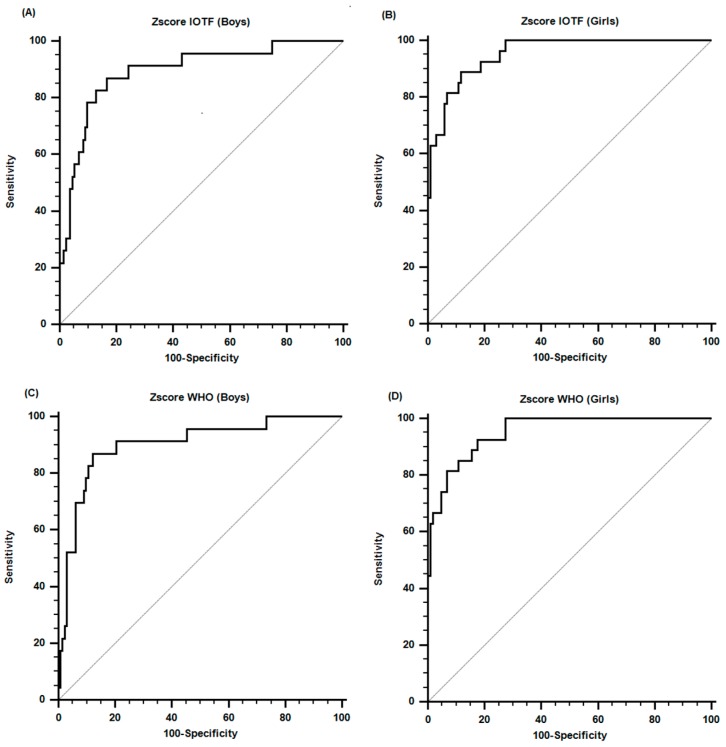
ROC curves of Z-scores of IOTF: (**A**) Boys; (**B**) Girl and WHO: (**C**) Boys; (**D**) Girl that showed the highest sensitivity and specificity when used in Chilean children and adolescents, using the cutoffs of the fat mass percentage values measured by DXA as criteria.

Table 3 shows the parameters of the ROC curve. It was verified by both references because they had the ability to predict obesity in both sexes. Figure 2 illustrates the ROC curves of Z-scores of boys and girls. The sensitivity values varied between 87% and 93% and the specificity from 83% to 88%, respectively.

## 4. Discussion

In this study focusing on Chilean children and adolescents, BMI Z-score values from international references of the WHO [13] and the IOTF [12] were highly correlated with %FM measured by the DXA. However, the prevalence of obesity classified by various cut-off points was different (see Table 2). What this suggests is that in this sample, the cut-off points to determine obesity should be chosen with caution.

The BMI cut-off points established by the IOTF were obtained from data from six countries (Brazil, Great Britain, Hong Kong, The Netherlands, Singapore, and the United States) collected between 1963 and 1993. Moreover, from the ages of 2 to 18 years (except for Singapore commencing at age 6) and separated by sex, the data was categorized as overweight (25 kg/m^2^), obese (30 kg/m^2^), and underweight (level 1: 18.5 kg/m^2^; level 2: 17.0 kg/m^2^, and level 3: 16.0 kg/m^2^).

The BMI cut-offs developed by Cole and Lobstein [12] were established due to their ease of use and comparisons with other references. The values of those references may be expressed in percentiles or Z-scores. In fact, this last calculation is derived by using the formula and LMS method where L stands for the asymmetry of σ, M refers to the median of λ, and S stands for the coefficient variation of *μ* [12].

Another reference used was that of the WHO. This one was created with the goal of evaluating the growth of children and adolescents. The data from the NCHS/OMS relates to 1997. It was revised in 2007 with the *Health Examination Survey* (HES). In Cycle II, it was composed of children ages 6 to 11 years old and in Cycle III with adolescents 12 to 17 years of age. Cycle I included data from birth to 74 months old. In general, the data was collected using the *Health and Nutrition Examination Survey* (NHANES). This survey used data from age 1 to 24 years old. The new growth curves were created using standards from the WHO (from 0 to 5 years of age) and the NCHS (18 to 71 months old). To sum up, the total sample included 22,917 subjects representing the growth curves for the population of 18 months to 24 years of age [13].

In essence, despite the fact that the BMI does not distinguish the components of body composition, (values for body fat and body mass free from fat), the values of the BMI Z-scores demonstrated that they were a good indicator of body fat in children and adolescents in this study. In fact, the highly correlated values that resulted are demonstrated in Figure 1 between the %FM and the BMI Z-scores (calculated with two references). These results were also found in other studies based on research of healthy children [20,21] or with some diseases particularly like diabetes [22] and cerebral palsy [23].

On the other hand, in associating the percentage of DXA body fat with the Z-scores from the WHO [13] and the IOTF [12], the first reference [13] identified more adolescents as obese.

The fact that the cut-off points for the Z-scores established for obesity (percentile 97) for both sexes is +2.0 leads us to believe that these values may be overestimated. Therefore, perhaps, a better evaluation and classification of nutrition needs to be undertaken. However, the IOTF [12] classification is +2.3 for boys and +2.2 for girls. The WHO reference always contains more subjects in the category of obese when compared to the IOTF. This presents values closer to the general percentages of individuals classed as obese by the DXA reference.

Therefore, various studies indicate that the relationship between the increase in BMI and the risk of developing illnesses such as cardiovascular, diabetes, hypertension, dyslipidemia, and obesity, among others [24,25]. In this sense, children and adolescents with BMI equal to or greater than the 85th percentile are characterized by the WHO [18], as overweight. This cut-off point allows the prediction of risk factors to health, such as obesity, that is an epidemic for all age groups. It also carries the risk of morbidity, and mortality, and even reduced life expectancy [6].

From this perspective, another study carried out by Rudolf, Krom, Cole [26] with 54 children and adolescents between the ages of 8 to 15 for 6 months verified that the measurements carried out by the BMI Z-scores showed less correlation with body fat in relation to those conducted with the DXA. However, this is the best indicator to measure body fat. A small discrepancy was observed in the BMI Z-score measurements carried out by different methods. From this perspective, it can be suggested that the BMI may be a useful tool in controlling weight and as a reasonable indicator of body fat.

In general, this present study determined cut-off points for the BMI Z-scores for boys and girls. Based on the measures obtained from the DXA measurements and in keeping with the IOFT classification [12], cut-off points for boys and girls were 1.0 and 1.8 (see Table 3). The sensitivity for the cut-off point for boys was 87%. This means that starting with this reference for obese boys, 87% of them were diagnosed correctly as obese compared to the DXA reference. Specificity was 84%. When this is used as a reference, 84% were correctly diagnosed as not obese. As a result, this same interpretation may be used in the case of the girls (89% for sensitivity and 88% for specificity) in this study since relatively similar values occurred with respect to the boys.

The Z-score classification by the WHO [13] was used. The cut-ff point for obesity for boys was 2.3 and 1.6 for girls (Table 3). As stated before, these atypical values were produced since the two references used in this study had different cut-off points for obesity. In this respect, Farías Junior *et al.* [27] examined five classification criteria for BMI proposed by Must, Dallal, and Dietz [14], the WHO [28], the IOTF [29], the CDC-2002 [30], and Conde and Monteiro [31]. Farías Junior *et al.* [27] found low sensitivity values in girls (less than 52.8%) and elevated values in boys (greater than 85%) just as in our study depicted in Table 3. This clearly coincides with our research.

As a result, the high sensitivity values observed in the Chilean girls may be basically the result of their presenting elevated BMI values when compared to the young Americans [13]. In fact, the differences between both sexes could be due to genetic, hormonal, cultural, and environmental differences. However, generally, the majority of the variations occur during puberty where girls have more body fat in relation to boys while boys have a greater muscle mass during this time [32]. Another question to be discussed in this study is that the body composition changes that occur during sexual maturation identified by the BMI since the final result is a function of total weight gain (without taking into account body fat and muscle mass values).

In summary, in the search for eequilibirum between specificities, the IOFT [12] is considered to be an accurate tool for predicting obesity starting from the BMI. The predictive values were high, and they approximated the specificity values in comparison to the NHANES [20].

One limitation of this study is the lack of control for biological maturation. This factor could possibly affect the findings, particularly the proposed cut-off points, taking into account the hormonal changes that occur during growth and development in children and adolescents. An easy alternative to use and implement could be the evaluation of the duration of somatic maturation based on anthropometric measurements as s proposed by Cossio-Bolaños *et al.* [33].

## 5. Conclusions

In this sample of Chilean children and adolescents, the BMI Z-scores were calculated using the references from the WHO and the IOTF. They showed a high correlation with the %FM determined by the DXA. Furthermore, cut-off from both references demonstrated high for sensitivity and specificity values. However, the classifications used for the different BMI cut-off points showed discrepancies. This suggests that the selection of the cut-off points for obesity should be viewed with caution. Determining a suitable cut-off point is fundamental for identifying obesity in populations in order to implement policies to prevent non-transmittable chronic illnesses.

## Figures and Tables

**Table 1:** Characteristics of sample of Chilean children and adolescents by sex.

Variables	TOTAL; *n* = 280	BOYS; *n* = 155	GIRLS; *n* = 125
	Median (Minimum–Max)	Median (Minimum–Max)	Median (Minimum–Max)
AGE (years)	11.2 (8.0–16.9)	11.3 (11.3–16.9)	11.1 (8.0–15.2)
WEIGHT (kg)	45.6 (19.9–88.3)	45.4 (20.0–82.0)	45.7 (19.9–88.3)
HEIGHT (cm)	145.3 (120.0–177.0)	145.7 (120.0–177.0)	143.4 (120.0–166.0)
BMI (kg/m^2^)	21.1 (12.0–38.2)	20.9 (12.0–31.6)	21.3 (12.5–38.2)
%FM DXA	32.8 (13.9–51.7)	30.7 (13.9–46.4)	35.4 (17.6–51.7)
Z-SCORE IOTF (BMI)	1.3 (−3.1–3.3)	1.2 (−3.1–3.3)	1.5 (−2.3–3.2)
Z-SCORE WHO (BMI)	1.2 (−3.7–5.1)	1.3 (−3.7–5.1)	1.2 (−2.4–3.3)

Max: Maximum; BMI: Body Mass Index; %FM DXA: percent of fat mass by dual energy X-ray absorptiometry.

**Table 2:** Values of the prevalence of obesity in Chilean children and adolescents estimated by using the %FM determined by DXA and the Z-scores based on the IOTF and the WHO references.

BMI Cut Off	Category	%FM DXA							
		Boys *n* = 155				Girls *n* = 125			
		Normal *n* (%)	Obese *n* (%)	Total	*p*	Normal *n* (%)	Obese *n* (%)	Total	*p*
**IOTF**	**Normal**	121 (93.1)	9 (6.9)	130	<0.001	95 (91.3)	9 (8.7)	104	<0.001
	**Obese**	11 (44.0)	14 (56.0)	25		3 (14.3)	18 (85.7)	21	
**WHO**	**Normal**	101 (98.1)	2 (1.9)	103	<0.001	90 (94.7)	5 (5.3)	95	<0.001
	**Obese**	31 (59.6)	21 (40.4)	52		8 (26.7)	22 (73.3)	30	
**Total**		132 (85.2)	23 (14.8)			98 (78.4)	27 (21.6)		

Abbreviations: %FM DXA: percent of fat mass by Dual Energy Absorptiometry X-ray; BMI: Body Mass Index.

**Table 3:** Descriptive values of the ROC Curve in both sexes.

References	BOYS				GIRLS			
	CRITERION	AUC (IC 95%)	SENS (%)	SPEC	CRITERION	AUC (IC 95%)	SENS (%)	SPEC
**IOTF**	>1.9	0.89 (0.83–0.94)	87.0	83.3	>1.7	0.95 (0.90–0.98)	88.9	87.8
**WHO**	>2.3	0.90 (0.84–0.94)	86.9	87.9	>1.6	0.95 (0.90–0.98)	92.6	82.7

Abbreviations: AUC: Area under the curve; SENS: Sensibility; SPEC: Specificity; CRITERION: the cutoff values (Z-score) of international references (WHO and IOTF) that showed the highest sensitivity and specificity when used in Chilean children and adolescents, using the cutoffs of the fat mass percentage values measured by DXA as criteria.
